# The Role of *VEGFA* in T2DM-Nephropathy: A Genetic Association Study and Meta-Analysis

**DOI:** 10.3390/genes16111386

**Published:** 2025-11-17

**Authors:** Maria Tziastoudi, Christos Cholevas, Constantinos Zorz, Efthimios Dardiotis, Evangelia E. Tsironi, Maria Divani, Theodoros Eleftheriadis, Ioannis Stefanidis

**Affiliations:** 1 Department of Nephrology, Faculty of Medicine, University of Thessaly, 41110 Larissa, Greece; mkzorz@yahoo.gr (C.Z.); mariadivani@yahoo.com (M.D.); teleftheriadis@uth.gr (T.E.); stefanid@uth.gr (I.S.); 2 Laboratory of Pharmaceutical Technology, Division of Pharmaceutical Technology, School of Pharmacy, Faculty of Health Sciences, Aristotle University of Thessaloniki, 54124 Thessaloniki, Greece; ccholevas@auth.gr; 3 Department of Neurology, University Hospital of Larissa, Faculty of Medicine, School of Health Sciences, University of Thessaly, 41110 Larissa, Greece; edar@uth.gr; 4 Department of Ophthalmology, Faculty of Medicine, School of Health Sciences, University of Thessaly, 41110 Larissa, Greece

**Keywords:** diabetes mellitus, diabetic nephropathy, vascular endothelial growth factor (*VEGFA*), genetic association study, meta-analysis

## Abstract

**Background**: Diabetic nephropathy (DN) is a leading cause of end-stage renal disease, arising from complex interactions between metabolic, hemodynamic, and genetic factors. Among candidate genes, vascular endothelial growth factor A (*VEGFA*) has been extensively investigated due to its role in endothelial homeostasis and microvascular complications of diabetes. The present study aimed to examine the association of *VEGFA* polymorphisms with DN in a Greek population and to perform a comprehensive meta-analysis of available evidence. **Methods**: A case–control study was conducted, including 197 patients with type 2 diabetes mellitus (T2DM) and DN, 155 diabetic patients without nephropathy, and 246 healthy controls. Ten tagging single-nucleotide polymorphisms (SNPs) across *VEGFA* were genotyped. Statistical analyses employed the generalized odds ratio (OR_G_). To contextualize these findings, a meta-analysis of 13 eligible studies was performed, encompassing 7520 cases, 6951 diabetic controls, and 1718 healthy controls. **Results**: Of the tested variants in the present case–control study, only rs833070 was significantly associated with DN across all comparisons. Nine *VEGFA* variants were evaluated in meta-analysis, with rs2146323 showing a protective effect (allelic OR = 0.85; 95% CI: 0.76–0.95), while other variants yielded non-significant associations. **Conclusions**: Overall, the data suggest that *VEGFA* polymorphisms, particularly rs833070 and rs2146323, contribute to genetic susceptibility to DN, although population-specific differences and heterogeneity across studies remain substantial. Future research in large, ethnically diverse cohorts with functional analyses is warranted to clarify causal mechanisms and enable the integration of *VEGFA* genetic variation into risk stratification and personalized therapeutic strategies.

## 1. Introduction

Diabetic nephropathy (DN) is a progressive microvascular complication associated with both type 1 and type 2 diabetes and continues to be the primary cause of end-stage renal disease (ESRD) globally, affecting approximately 30–40% of individuals with diabetes [1]. It is clinically defined by persistent micro/macroalbuminuria, a sustained decline in estimated glomerular filtration rate (eGFR), and often hypertension, confirmed on at least two occasions over 3–6 months [2]. Pathophysiologically, the disease is driven by hyperglycemia-induced metabolic dysregulation (e.g., advanced glycation end-products), hemodynamic changes (glomerular hyperfiltration, RAAS activation), and inflammatory and profibrotic cytokine signaling, resulting in glomerular basement membrane thickening, mesangial expansion, podocyte injury, and eventual fibrosis [3,4]. Major risk factors include poor glycemic control, uncontrolled hypertension, dyslipidemia, obesity, smoking, family history, and certain ethnic predispositions [5]. Early intervention—comprising strict glucose and blood pressure control, renin-angiotensin system blockade, SGLT2 inhibitors, GLP-1 receptor agonists, and lifestyle modification—can delay progression and improve renal and cardiovascular outcomes [6,7].

Although glycemic and hemodynamic disturbances play a central role, the genetic contribution to DN is undeniable yet remains poorly understood [8]. Multiple genetic loci have been associated with the disease’s pathogenesis; however, each locus exerts only a modest effect size [9]. Different methodological approaches have been implemented. Among them, linkage studies [10,11] and genetic association studies [12,13] are the most common study designs, as well as meta-analyses of these studies [14,15,16].

Vascular endothelial growth factor A (*VEGFA*) is a crucial regulator of endothelial cell proliferation, differentiation, and survival, and is essential for both physiological and pathological angiogenesis [17,18]. In the healthy kidney, podocytes secrete *VEGFA* to support the integrity and function of glomerular and peritubular endothelial cells, helping maintain the filtration barrier [19,20]. However, in diabetes, local *VEGFA* expression becomes dysregulated, as rodent models of diabetic nephropathy show elevated renal *VEGFA*, and pharmacological blockade of VEGF signaling in these models reduces albuminuria and glomerular injury [21,22]. More specifically, diabetes is associated with increased levels of glomerular VEGFA expression [23]. However, human data present a nuanced picture, as some studies find higher circulating VEGF associated with worse albuminuria and glycemic control, while others find no or even inverse relationships, suggesting that timing, isoform, receptor context, and disease stage critically shape VEGF’s impact in diabetic nephropathy [24].

Several genetic association studies (GAS), as well as many meta-analyses, have implicated certain *VEGFA* variants in the pathophysiology of DN, but the results are inconsistent. For instance, the promoter variant rs833061 (−460 T/C) was associated with DN in a meta-analysis of two studies (*n* = 543) containing only type 1 diabetes mellitus patients of European origin [25]. A broader meta-analysis covering multiple *VEGFA* variants also identified rs833061 as significantly associated with DN risk across diverse populations [26]. In a large case–control study among Han Chinese type 2 diabetic patients, *VEGFA* rs2010963 and rs69947 variants were linked to increased DN susceptibility [27]. These findings support the hypothesis that common functional *VEGFA* polymorphisms modulate *VEGF* expression and microvascular vulnerability in diabetes, although effects vary by ancestry, diabetes type, and study design—highlighting the need for larger, ethnically diverse cohorts and deeper functional validation. Another variant across *VEGFA* that has been examined in meta-analyses is rs2146323 [14,16,28].

Given the pivotal role of *VEGFA* in angiogenesis and glomerular endothelial function, genetic variations within this gene are strong candidates for influencing susceptibility to diabetic nephropathy. However, findings from previous studies remain inconsistent, likely due to differences in study design, sample size, ethnicity, and diabetes type. To address these gaps, we conducted a case–control association study in a well-characterized Greek cohort, genotyping ten tagging SNPs across the *VEGFA* gene. In parallel, we performed a systematic review and meta-analysis of all available genetic data on *VEGFA* variants and DN, thereby providing the most comprehensive evaluation to date. It is noteworthy to mention that the odds ratio generalized (OR_G_) will be used for the analysis of genotypic data in the context of a genetic model-free approach. This combined approach aims to clarify the contribution of *VEGFA* genetic variation to DN risk and progression, with the ultimate goal of improving understanding of disease pathogenesis and guiding future precision medicine strategies.

## 2. Materials and Methods

### 2.1. Association Study

#### 2.1.1. Subjects

Comprehensive information on the study design and demographic characteristics of the participants has been reported previously [29]. Briefly, the study included 197 patients with DN, 155 diabetics (T2DM) but without microvascular complications, and 246 healthy controls. The participants were evaluated at the Ophthalmology and Nephrology clinics of the University Hospital of Larissa, Greece.

DN was defined as persistent macroalbuminuria, with urinary albumin excretion > 300 mg/24 h (>200 μg/min), irrespective of serum creatinine levels. The study protocol was approved by the Ethics Committee of the University of Thessaly, and written informed consent was obtained from all participants prior to enrollment.

#### 2.1.2. Genotyping

Genotyping was performed as has been reported previously [29]. A total of 10 tag SNPs across *VEGFA* were retrieved (rs3025053, rs3025040, rs10434, rs25648, rs3024994, rs3025035, rs2146323, rs3024997, rs2010963, rs833070). The selection of tagging SNPs was performed using an r^2^ threshold of ≥0.8 and a minor allele frequency (MAF) greater than 0.05. To ensure accuracy, at least 10% of the samples were randomly selected for repeat genotyping as an internal quality control. All genotyping was conducted by staff blinded to the participants’ clinical status.

#### 2.1.3. Data Analysis

The generalized odds ratio (OR_G_) was used for the assessment of the association between genotype distribution and disease risk [30,31]. In healthy controls, genotype distributions were also tested for deviation from Hardy–Weinberg equilibrium (HWE). The OR_G_ was calculated with the ORGGASMA software (http://biomath.med.uth.gr; accessed 30 August 2025) [30,31]. All statistical analyses were performed using SPSS version 29.0 for Windows (SPSS 29.0 Inc., Chicago, IL, USA).

### 2.2. Meta-Analysis

All studies published up to August 2025 were identified through a comprehensive PubMed search. The following search terms were applied: [‘diabetic nephropathy’ AND ‘association’ AND (‘*VEGFA*’ OR ‘vascular endothelial growth factor’)]. In addition, relevant records were retrieved from the Genome-Wide Association Studies (GWAS) Catalog (https://www.ebi.ac.uk/gwas/; accessed 30 August 2025). Case reports, editorials, and review articles were excluded. The search was restricted to English-language publications.

Eligible studies for inclusion in the meta-analysis were case–control designs that investigated *VEGFA* gene polymorphisms in patients with DN compared with either diabetic controls without DN or healthy controls. The inclusion criteria for DN cases and both control groups were consistent with those applied in the present association study. Genome linkage scans were excluded, as they represent a different study design.

From each eligible study, the following data were extracted: first author, year of publication, ethnicity, type of diabetes, and phenotype. For both cases and controls, we recorded sample size and selection criteria. Genotypic information was extracted as complete genotype counts or allele frequencies.

For meta-analysis, a minimum of two studies per genetic variant was required. Pooled ORs were calculated with the DerSimonian and Laird random-effects model [32]. Between-study heterogeneity was assessed using Cochran’s Q test [33] and quantified with the *I*^2^ statistic [34].

## 3. Results

### 3.1. Association Analysis

The study population included 197 cases with DN (DM+DN), 155 diabetics without DN (DN-DN), and 246 healthy controls (HC). All participants were Caucasians of Greek origin. Demographic data and clinical profile are summarized in Table 1, as have also been described elsewhere [35]. Among the 197 DN cases, 11 had progressed to end-stage renal disease (ESRD).

The genotype distributions of the ten variants (rs3025053, rs3025040, rs10434, rs25648, rs3024994, rs3025035, rs2146323, rs3024997, rs2010963, rs833070) and the respective OR_G_ are shown in Table 2, Table 3 and Table 4. Only rs833070 was found statistically significant [OR_G_ = 1.26, 95% CI (1.01, 1.59)] in healthy controls versus diseased controls versus cases and in diseased controls versus cases [OR_G_ = 1.46, 95% CI (1.01, 2.12)], as well as in healthy controls versus cases [OR_G_ = 1.43, 95% CI (1.03, 1.99)].

### 3.2. Meta-Analysis

**Figure 1:**
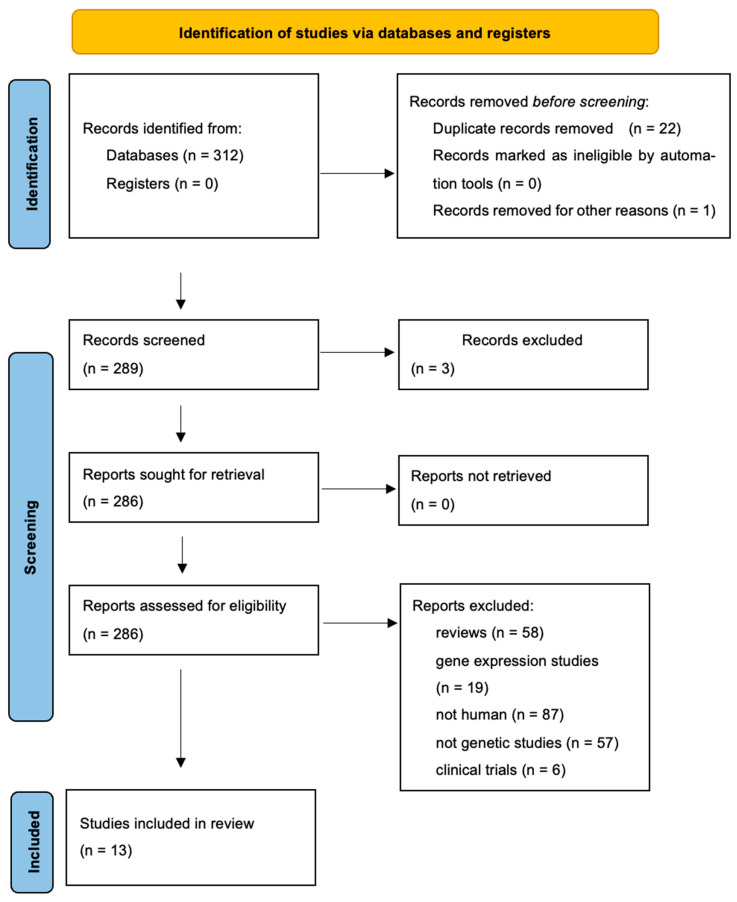
Flowchart showing how studies were selected for meta-analysis.

The literature search yielded 312 PubMed records that met the inclusion criteria. When a study reported data from different populations, each population was treated as a separate dataset. A flowchart summarizing the selection process and reasons for exclusion is provided in Figure 1, while the characteristics of the included studies are detailed in Table 5.

**Figure 2:**
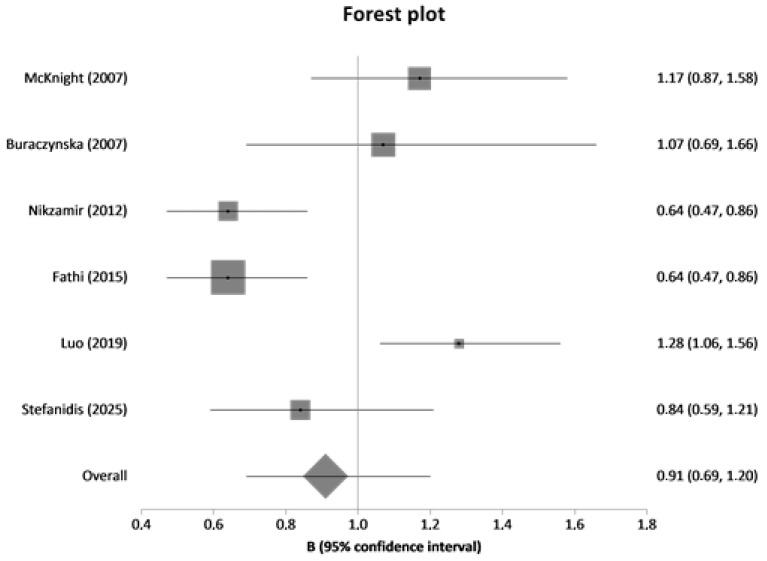
Forest plot of rs2010963 between diseased controls (diabetics without DN) versus cases (diabetics with DN).

In meta-analysis, nine variants (rs2010963, rs699947, rs833061, rs35569394, rs6921438, rs10738760, rs2146323, rs3024997, rs3025000) were included. The studies were published between 2003 and 2024. The forest plot is presented in Figure 2. Meta-analysis results are presented in Table 6 and Table 7.

## 4. Discussion

While hyperglycemia and hypertension are recognized as crucial pathogenic factors, growing evidence highlights the multifactorial nature of DN, with genetic predisposition playing a significant role in susceptibility and progression [47]. Among the candidate genes, *VEGFA* has attracted considerable attention due to its established involvement in diabetic microvascular complications [20,48,49]. Genetic variations in *VEGFA* are believed to influence the risk of developing DN, although findings vary among studies and populations [14]. To provide a comprehensive assessment of genetic variation in the *VEGFA* gene, we selected ten tag SNPs for genotyping in a Greek-origin cohort and conducted a systematic review and meta-analysis incorporating all available genetic data on variants of this gene, providing the most comprehensive overview assessing the contribution of *VEGFA* variants in DN risk and progression.

Out of ten variants, only rs833070 was revealed statistically significant in all analyses in the context of the present GAS. From these variants, available data from other studies were only for rs2146323, rs3024997, and rs2010963. In agreement with our findings, Trégouet et al. did not find statistically significant differences regarding rs2146323 and rs3024997 [46]. Findings from the Ensembl genome browser suggest that rs833070, which is an intron variant, is positioned within a predicted regulatory element enriched in CTCF and DNase I binding sites, which may influence *VEGF* expression levels [50]. This observation was further supported by a second bioinformatics analysis using FastSNP [51,52].

Regarding rs2010963, the results of previous studies are conflicting. In Iranian T2DM patients, both the GG genotype and G allele of the VEGF +405 G/C polymorphism were significantly more frequent in patients with microalbuminuria (indicative of DN) times [39]. More specifically, the GG genotype was an independent predictor, increasing the risk of microalbuminuria by 2.22 times [39]. Another Iranian study confirmed the GG genotype as an independent predictor for albuminuria (*p* = 0.014, OR = 1.771), also noting that glomerular filtration rate (GFR) varied significantly by genotype [36]. For Han Chinese individuals, the GC/CC genotypes at rs2010963 were also significantly associated with an increased risk of T2DN (OR = 1.15), and wild-type VEGFA protein levels were higher than mutant types [27]. In contrast, a Polish study and an Irish study found no significant association between this polymorphism and DN [38]. Similarly, a separate study failed to find an association between this variant and diabetic nephropathy [37]. It has been found that the C allele of rs2010963 was associated with increased *VEGFA* expression, suggesting that rs2010963 functions as an eQTL for the VEGFA gene and can affect the gene expression of VEGFA in multiple normal human tissues [53].

The findings of a study indicate that the I/D polymorphism in the VEGF gene promoter is not linked to DN in West Indian patients with type 2 diabetes, but it may contribute to the development of non-diabetic nephropathy [42]. Another study in the Indian population with type 2 diabetes (T2DM), the DD genotype and D allele of the 18 bp I/D polymorphism at −2549 position were significantly associated with DN, conferring a 4.2-fold increased risk for the DD genotype and a 2.2-fold increased risk for the D allele [43]. The D allele is hypothesized to be associated with increased transcription of VEGFA. Similarly, a study on Type 1 diabetes (T1DM) patients in the UK observed a significantly increased frequency of the D/D genotype (40.2% vs. 22.7%) and the D allele in patients with nephropathy compared to uncomplicated diabetic patients [44]. Functional studies indicated that the construct containing the 18 bp deletion had a 1.95-fold increase in transcriptional activity. Conversely, a Polish study in T2DM patients found no significant association of the DD genotype or D allele with DN. However, this study did observe a significant association between the DD genotype and diabetic retinopathy and suggested an inadequate sample size for the DN analysis [38].

In addition, despite their influence on circulating VEGF levels, Bonnefond et al. reported no association between SNPs rs6921438 and rs10738760 and the risk of type 2 diabetes, diabetic nephropathy, or retinopathy [44]. However, this study reported an association between the G-allele of rs6921438 (linked to higher circulating VEGF levels) and increased T2DM risk and higher HbA1c in the French population, an observation not replicated in the Danish cohort [44]. However, in Slovenian patients with type 2 diabetes, the G allele of rs6921438-VEGF was associated with a reduced risk of developing diabetic nephropathy [45]. More specifically, individuals with the G allele (GG+AG genotypes) had a 0.66-fold lower risk of DN.

Regarding VEGF-1499 C/T polymorphism (rs833061), an Irish study on T1DM patients found a positive association between the VEGF-1499T allele and DN, which was replicated in an independent population. Carriage of the T allele was associated with a 2.24-fold increased risk of developing DN [37].

This study has several strengths, including the combined use of a case–control association study and a comprehensive meta-analysis, which together provide robust insights into the contribution of VEGFA variants to diabetic nephropathy (DN). The use of a well-characterized Greek cohort, systematic selection of tag SNPs, and rigorous statistical methods further enhance the reliability of the findings.

Nonetheless, some limitations should be acknowledged. The relatively modest sample size may have reduced statistical power for detecting small effect sizes, and the restriction to a single ethnic group limits generalizability. In addition, the lack of multiple-testing correction entails the risk significant association of rs833070 is a false-positive result. Regarding the meta-analysis, heterogeneity across studies in ethnicity, diabetes type, and DN diagnostic criteria complicates interpretation. Moreover, the lack of functional validation prevents mechanistic conclusions, and the cross-sectional design of the association study precludes causal inferences. Future research in larger, multi-ethnic cohorts with functional analyses is warranted to clarify the biological role of VEGFA variants in DN.

## 5. Conclusions

In conclusion, while the precise mechanisms require further elucidation, the data suggest that *VEGFA* polymorphisms, particularly rs833070 and rs2146323, contribute to genetic susceptibility to DN. The genetic model-free approach takes advantage of the full genotype distribution and provides a straightforward interpretation of genetic associations. However, future research should aim for larger, multi-ethnic, prospective cohort studies with detailed phenotypic characterization and functional validation to clarify the impact of these genetic variants, individually and in combination with other genetic or environmental factors. Such comprehensive efforts could ultimately enable the identification of individuals at increased risk for DN, paving the way for targeted screening and personalized therapeutic interventions.

## Figures and Tables

**Table 1:** Clinical profiles of the study cohort.

Parameters	Case–Control Study Groups (*n* = 498)					
	HC	DM	*p* Value *	DM-DN	DM+DN	*p* Value *
N	246	352	n.a.	155	197	n.a.
Sex [m; *n* (%)]	136 (55.3)	181 (51.4)	0.361	74 (47.7)	107 (54.3)	0.238
Age (years)	71 ± 9.2	68 ± 8.9	<0.001	68 ± 9.1	69 ± 8.8	0.427
DM duration (years)	n.a.	16.3 ± 8.0	n.a.	15.7 ± 8.3	16.8 ± 7.8	0.508
HbA1c (%)	n.d.	7.35 ± 1.31	n.a.	7.20 ± 1.34	7.47 ± 1.29	0.019
Insulin treatment (%)	n.a.	105 (29.8)	n.a.	50 (32.3)	55 (27.9)	0.412
Hypertension (%)	0	224 (63.6)	<0.001	98 (63.2)	126 (63.9)	0.911
Cardiovascular disease (%)	0	110 (31.3)	<0.001	41 (26.5)	69 (35.0)	0.105
Creatinine (mg/dL)	0.77 ± 0.15	1.46 ± 1.37	<0.001	0.90 ± 0.18	1.84 ± 1.67	<0.001
Urea (mg/dL)	30 ± 7.9	59 ± 34	<0.001	42 ± 13.6	71 ± 38.3	<0.001
Albuminuria (mg/d)	n.d.	470 ± 856	n.a.	43.9 ± 53.3	782 ± 1019	<0.001
Proteinuria (mg/d)	n.d.	788 ± 1468	n.a.	105 ± 80.0	788 ± 1468	<0.001

n.a.: not applicable, n.d.: not defined. * *p* values were calculated by the Mann–Whitney U test for continuous variables or the χ^2^ test for categorical variables as appropriate.

**Table 2:** Association between *VEGFA* variants and T2DM-nephropathy in healthy cases versus diseased controls versus cases.

VEGFA	Alleles	HT	DC	DN	OR_G_
rs3025053	GG	201	133	170	0.84 (0.56, 1.24)
	GA	34	20	22	
	AA	0	0	0	
rs3025040	CC	173	97	138	0.92 (0.65, 1.31)
	CT	60	50	50	
	TT	8	4	4	
rs10434	AA	51	28	43	0.96 (0.73, 1.27)
	AG	123	77	11	
	GG	65	46	40	
rs25648	CC	178	115	133	1.15 (0.84, 1.57)
	CT	55	29	47	
	TT	5	2	8	
rs3024994	CC	227	145	189	0.65 (0.35, 1.21)
	CT	13	8	5	
	TT	0	0	0	
rs3025035	CC	210	132	166	1.00 (0.67, 1.49)
	CT	33	17	27	
	TT	1	0	0	
rs2146323	CC	105	73	88	1.01 (0.79, 1.29)
	CA	108	70	84	
	AA	19	8	20	
rs3024997	GG	81	55	69	0.92 (0.73, 1.15)
	GA	111	63	95	
	AA	44	31	28	
rs2010963	CC	44	32	28	1.12 (0.90, 1.40)
	CG	120	68	97	
	GG	79	53	70	
rs833070	GG	84	57	52	1.26 (1.01, 1.59)
	GA	121	69	105	
	AA	34	24	38	

**Table 3:** Association between *VEGFA* variants and T2DM-nephropathy in diseased controls versus cases.

VEGFA	Alleles	DC	DN	OR_G_
rs3025053	GG	133	170	0.86 (0.45, 1.63)
	GA	20	22	
	AA	0	0	
rs3025040	CC	97	138	0.71 (0.46, 1.11)
	CT	50	50	
	TT	4	4	
rs10434	AA	28	43	0.78 (0.50, 1.20)
	AG	77	11	
	GG	46	40	
rs25648	CC	115	133	1.55 (0.95, 2.54)
	CT	29	47	
	TT	2	8	
rs3024994	CC	145	189	0.50 (0.17, 1.48)
	CT	8	5	
	TT	0	0	
rs3025035	CC	132	166	1.25 (0.66, 2.36)
	CT	17	27	
	TT	0	0	
rs2146323	CC	73	88	1.19 (0.81, 1.76)
	CA	70	84	
	AA	8	20	
rs3024997	GG	55	69	0.90 (0.63, 1.30)
	GA	63	95	
	AA	31	28	
rs2010963	CC	32	28	1.18 (0.82, 1.70)
	CG	68	97	
	GG	53	70	
rs833070	GG	57	52	1.46 (1.01, 2.12)
	GA	69	105	
	AA	24	38	

**Table 4:** Association between *VEGFA* variants and T2DM-nephropathy in healthy controls versus cases.

VEGFA	Alleles	HT	DN	OR_G_
rs3025053	GG	201	170	0.77 (0.44, 1.36)
	GA	34	22	
	AA	0	0	
rs3025040	CC	173	138	0.98 (0.65, 1.47)
	CT	60	50	
	TT	8	4	
rs10434	AA	51	43	0.85 (0.56, 1.30)
	AG	123	11	
	GG	65	40	
rs25648	CC	178	133	1.25 (0.82, 1.89)
	CT	55	47	
	TT	5	8	
rs3024994	CC	227	189	0.49 (0.18, 1.35)
	CT	13	5	
	TT	0	0	
rs3025035	CC	210	166	1.02 (0.59, 1.75)
	CT	33	27	
	TT	1	0	
rs2146323	CC	105	88	1.02 (0.73, 1.44)
	CA	108	84	
	AA	19	20	
rs3024997	GG	81	69	0.88 (0.63, 1.22)
	GA	111	95	
	AA	44	28	
rs2010963	CC	44	28	0.80 (0.55, 1.17)
	CG	120	97	
	GG	79	70	
rs833070	GG	84	52	1.43 (1.03, 1.99)
	GA	121	105	
	AA	34	38	

**Table 5:** Characteristics of studies included in meta-analysis.

Variant	References	Ethnicity	DM	Trait	Ν	Selection Criteria	Ν	Selection Criteria	N	Selection Criteria	Analyses
rs2010963	Nikzamir (2012) [36]	Asians	T2DM	DN	255	pers. macr/ria	235	pers. norm/ria			DC-C
	McKnight (2007) [37]	Caucasians	T1DM	DN	242	pers. macr/ria	301	pers. norm/ria	400	Healthy controls	DC-C, HT-DC-C, HT-C
	Buraczynska (2007) [38]	Caucasians	T2DM	DN	245	pers. macr/ria	181	pers. norm/ria			DC-C
	Luo (2019) [27]	Asians	T2DM	DN	650	pers. macr/ria	580	pers. norm/ria			DC-C
	Fathi (2015) [39]	East Asians	T2DM	DN	255	pers. micr/ria	235	pers. norm/ria			DC-C
	current study	-	T2DM	DN	197	pers. macr/ria	155	pers. norm/ria	246	Healthy controls	DC-C, HT-DC-C, HT-C
rs699947	McKnight (2007) [37]	Caucasians	T1DM	DN	242	DN	301	Diabetics without DN	400	Healthy controls	DC-C
	Luo (2019) [27]	East Asians	T2DM	DN	650	DN	580	Diabetics without DN			DC-C
−1499 C > T (rs833061)	McKnight (2007) [37]	Caucasians	T1DM	DN	242	proteinuria > 0.5 g/24 h, DM ≥ 10 yrs and DR	301	DM ≥ 15 yrs, norm/ria, no anti-HT meds	400	non-diabetics	DC-C
	Tiwari (2009) [40]	Asian Indians	T2DM	Diabetic CRI	90	moderate CRI, pers. s. Cr ≥ 2 mg/dL, DM ≥ 2 yrs, DR	75	DM ≥ 10 yrs and s. Cr < 2 mg/dL			DC-C
	Tiwari (2009) [40]	Asian Indians	T2DM	Diabetic CRI	106	moderate CRI, s. Cr ≥ 2 mg/dL, DM ≥ 2 yrs, DR	149	DM ≥ 10 yrs and s. Cr < 2 mg/dL			DC-C
−2549 I/D (rs35569394)	Yang (2003) [41]	Caucasians	T1DM	DN	102	DM ≥ 10 yrs, pers. macroalbuminuria, retinopathy, without hematuria	66	DM ≥ 20 yrs without retinopathy or proteinuria	141	non-diabetics	DC-C, HT-DC-C, HT-C
	Buraczynska (2007) [38]	Caucasians	T2DM	DN	245	pers. macr/ria of whom 43% with DR	91	DM ≥ 10 yrs, no nephropathy	493	non-diabetics	DC-C, HT-DC-C, HT-C
	Dabhi (2015) [42]	Asians	T2DM	DN	102	pers. micr/ria or proteinuria	103	diabetics with norm/ria	143	non-diabetics	DC-C, HT-DC-C, HT-C
	Amle (2015) [43]	Asians	T2DM	DN	40	macr/ria	40	only diabetics	40	non-diabetics matched for age, gender	DC-C, HT-DC-C, HT-C
rs6921438	Bonnefond (2013)-D2NG Study [44]	Caucasians	T2DM		547	different stages of renal involvement	286	normoalbuminuria, DM ≥ 10 yrs			DC-C
	Bonnefond (2013)-Corbeil [44]	Caucasians	T2DM		683	stage of kidney disease higher than 2	561	normoalbuminuria, DM ≥ 10 yrs			DC-C
	Nussdorfer (2024) [45]	Caucasians	T2DM	DN	344	pers. micr/ria or macroalbuminuria	553	normoalbuminuria, DM ≥ 10 yrs			DC-C
rs10738760	Bonnefond (2013)-D2NG Study [44]	Caucasians	T2DM		547	different stages of renal involvement	286	normoalbuminuria, DM ≥ 10 yrs			DC-C
	Bonnefond (2013)-Corbeil [44]	Caucasians	T2DM		683	stage of kidney disease higher than 2	561	normoalbuminuria, DM ≥ 10 yrs			DC-C
rs2146323, rs3024997, rs3025000	Tregouet (2008)-Denmark [46]	Caucasians	T1DM		489	persistent macroalbuminuria	463	normoalbuminuria, DM ≥ 15 yrs			DC-C
	Tregouet (2008)-Finland [46]	Caucasians	T1DM		412	persistent macroalbuminuria	614	normoalbuminuria, DM ≥ 15 yrs			DC-C
	Tregouet (2008)-France [46]	Caucasians	T1DM		300	persistent macroalbuminuria	391	normoalbuminuria, DM ≥ 15 yrs			DC-C

**Table 6:** Results from meta-analyses based on genotype counts.

Diseased Controls Versus Cases										
Gene	Variant	RS	Studies (n)	Cases/Controls (n)	RE OR_G_	95% LL	95% UL	*I^2^* (%)	*P_Q_*	*P_E_*
VEGFA		rs2010963	6	1842/1595	0.92	0.67	1.27	83.59	<0.001	0.27
		All in HWE	6							
VEGFA		rs699947	2	892/881	1.32	0.96	1.82	68.90	0.07	-
		All in HWE	2							
VEGFA	−1499 C > T	rs833061	3	435/524	1.28	0.63	2.57	88.86	0.00	0.16
		All in HWE	3							
VEGFA	I/D-2549	rs35569394	4	489/300	1.34	0.85	2.12	66.93	0.03	0.11
		All in HWE	4							
VEGFA		rs6921438	3	1574/1400	1.03	0.74	1.43	85.27	0.001	0.15
		All in HWE	3							
VEGFA		rs10738760	2	1223/843	0.98	0.85	1.14	0	0.40	-
		All in HWE	2							
**Healthy controls versus cases**										
VEGFA	I/D-2549	rs35569394	4	489/817	1.18	0.78	1.77	70.91	0.02	0.23
		All in HWE								
VEGFA		rs2010963	2	736/440	0.99	0.73	1.24	50.93	0.15	-
		All in HWE								
**Healthy controls versus diseased controls versus cases**										
VEGFA	I/D-2549	rs35569394	4	489/300/817	1.11	0.83	1.47	69.3	0.02	0.1
		All in HWE	4							
VEGFA		rs2010963	2	736/244/440	1.00	0.80	1.25	48.65	0.16	-
		All in HWE								

**Table 7:** Results from meta-analyses based on allele counts.

Gene	Variant	RS	Studies (n)	Cases/Controls (n)	RE OR	95% LL	95% UL	*I^2^* (%)	*P_Q_*	*P_E_*
VEGFA	C > A	rs2146323	3	1176/1323	0.85	0.76	0.95	0.2		0.2
VEGFA	G > A	rs3024997	3	1176/1323	1.03	0.90	1.18	0.27		0.27
VEGFA	C > T	rs3025000	3	1176/1323	1.01	0.89	1.14	0.18		0.18

## Data Availability

The datasets used and/or analysed during the current study are available from the corresponding author on reasonable request.
